# A Case Report of Refractory Penile Squamous Cell Carcinoma Treated With Surgical Resection, Chemotherapy and External Beam Radiation Therapy

**DOI:** 10.7759/cureus.41358

**Published:** 2023-07-04

**Authors:** Perry Zong, Jay Garlapati, Steven Miller

**Affiliations:** 1 Department of Radiation Oncology, Wayne State University School of Medicine, Detroit, USA

**Keywords:** endocarditis, covid-19, metastatic penile cancer, radiation oncology, radiotherapy, penile cancer

## Abstract

Penile cancer is a rare cancer in developed nations, with metastatic disease seen in under 10% of cases at presentation. Treatment is multimodal and dependent on tumor stage, risk of recurrence, nodal involvement, and advancement of the disease. We report a case of a 49-year-old male with penile squamous cell carcinoma, who initially presented with a penile lesion but became lost to follow-up due to the coronavirus disease 2019 (COVID-19) pandemic. Therapies included surgical resection, chemotherapy, and external beam radiation therapy. His disease course was additionally complicated by its aggressive nature, poor response to first-line chemotherapy, and secondary nosocomial infections.

## Introduction

Penile carcinoma is a rare neoplasm, accounting for less than 1% of all malignancies in men in the United States, but the incidence is highest in developing countries such as Brazil, where penile carcinoma is the fourth most common tumor in men. Squamous cell carcinoma (SCC) accounts for almost all penile carcinomas and most commonly affects the glans and prepuce. The exact etiology is not well known, but SCCs may arise de novo or from penile intraepithelial neoplasia (PIN). Human papillomavirus (HPV) infections, most commonly strains 16 and 18, are one of the most significant risk factors. Other risk factors include phimosis, lichen sclerosis, inflammatory conditions (balanitis), smoking, obesity, poor hygiene, lack of circumcision, and an increasing number of sexual partners [1-3]. 

We present a case of HPV-positive, metastatic invasive penile SCC which necessitated extensive treatment planning and administration. This tumor displayed many features characteristic of penile carcinomas, but a late presentation due to the coronavirus disease 2019 (COVID-19) pandemic led to metastases and a challenging treatment course. The patient underwent surgery, chemotherapy, and radiation therapy (RT) with varying levels of success. This case demonstrates the role of these treatment procedures and the impact they had on our patient. 

## Case presentation

**Figure 1:**
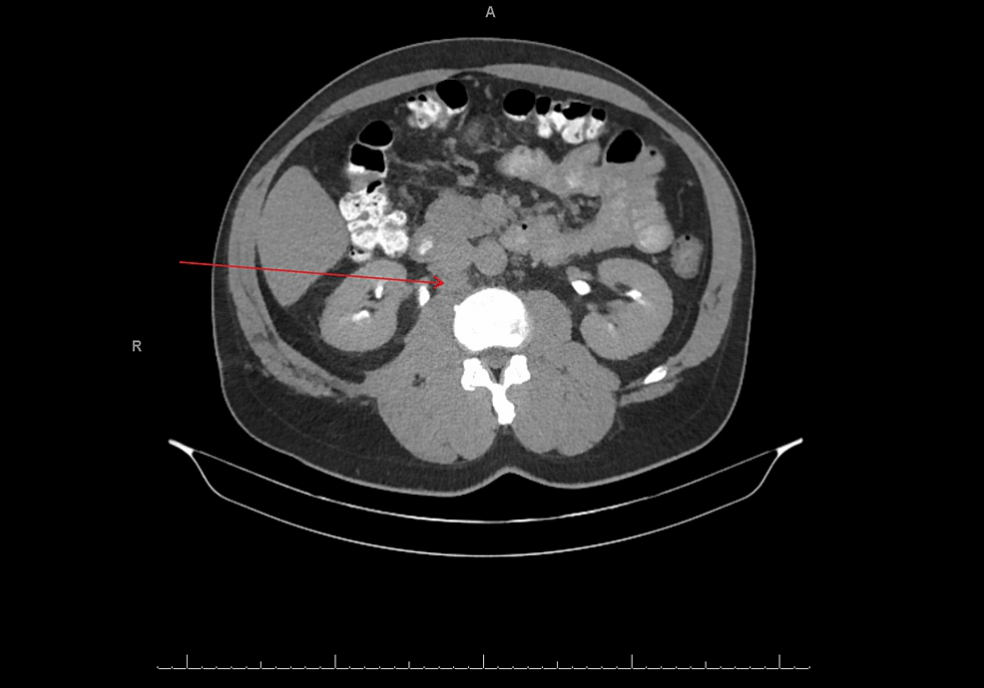
Pre-chemotherapy CT abdomen/pelvis with contrast showing retroperitoneal lymphadenopathy Arrow pointing at enlarged para-aortic lymph node.

**Figure 2:**
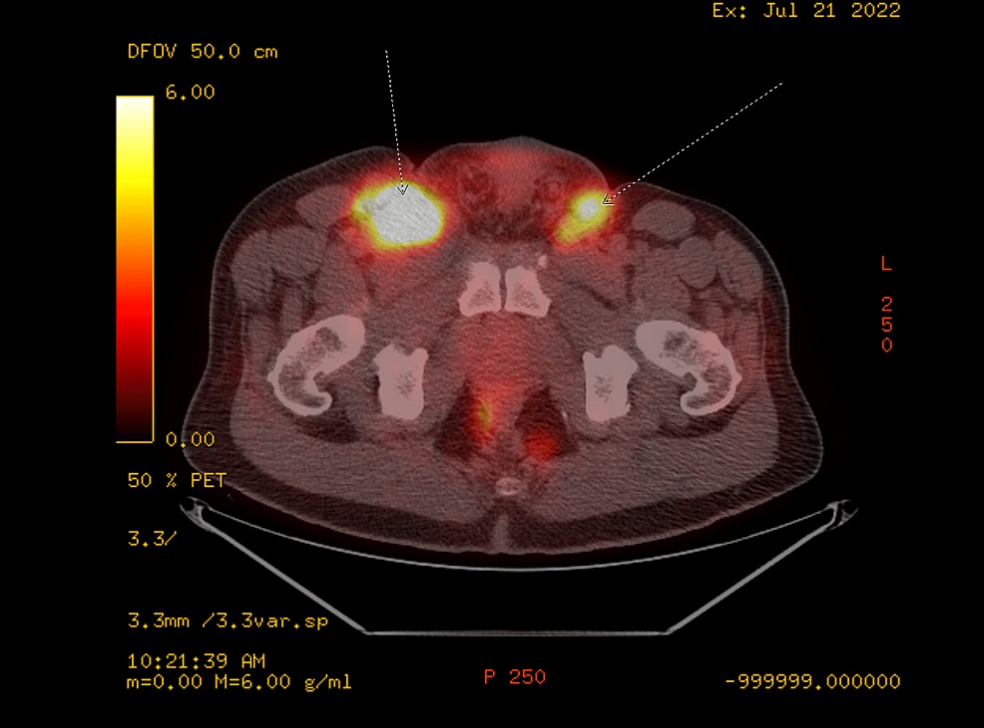
Pre-chemotherapy PET/CT demonstrating bilateral inguinal involvement Arrows pointing at areas of hypermetabolic activity in metastatic inguinal lymph nodes

A 49-year-old male with history of insulin-dependent diabetes mellitus, hypertension, and HPV, initially detected a penile lesion in November 2019, and underwent out-patient evaluation with urology. He was then given an appointment for biopsy, but he unfortunately was lost to follow-up just as the COVID-19 pandemic was starting up in early 2020. Over two years later, in February 2022, the patient presented to the emergency department because of painful penile swelling and he was subsequently admitted for further evaluation. A circumferential lesion was noted around the circumcised glans penis with friable tissue and penile edema. There were also findings of bilateral mobile inguinal lymphadenopathy. Urology performed an excisional biopsy of the meatal lesion, cystourethroscopy with urethral biopsy, and meatoplasty. Pathology results were consistent with low-grade superficial invasive squamous cell carcinoma. Further work-up was performed, including CT chest/abdomen/pelvis in May 2022 which revealed enlarged bilateral femoral and inguinal lymph nodes as well as enlarged right iliac chain lymph nodes (Figure 1). PET/CT scan in July 2022 showed multiple enlarged bilateral intensely fluorodeoxyglucose (FDG)-avid retroperitoneal, pelvic, inguinal, and femoral lymph nodes, compatible with metastatic disease (Figure 2).

**Figure 3:**
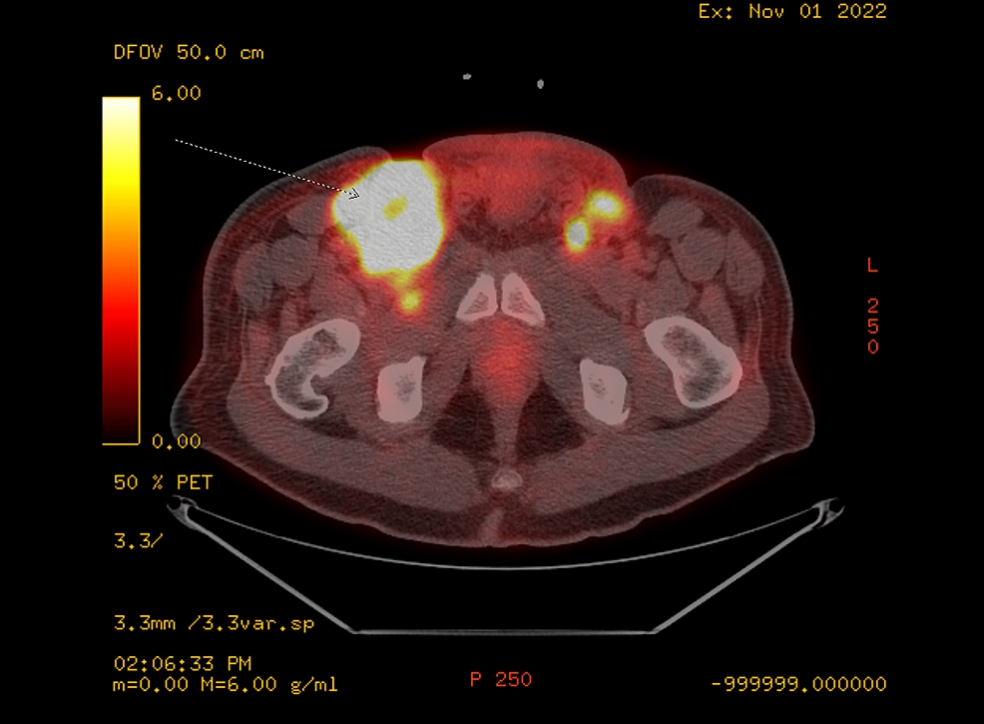
Post-chemotherapy PET/CT demonstrating disease progression in inguinal regions Arrow pointing at bulky refractory mass in right inguinal region.

**Figure 4:**
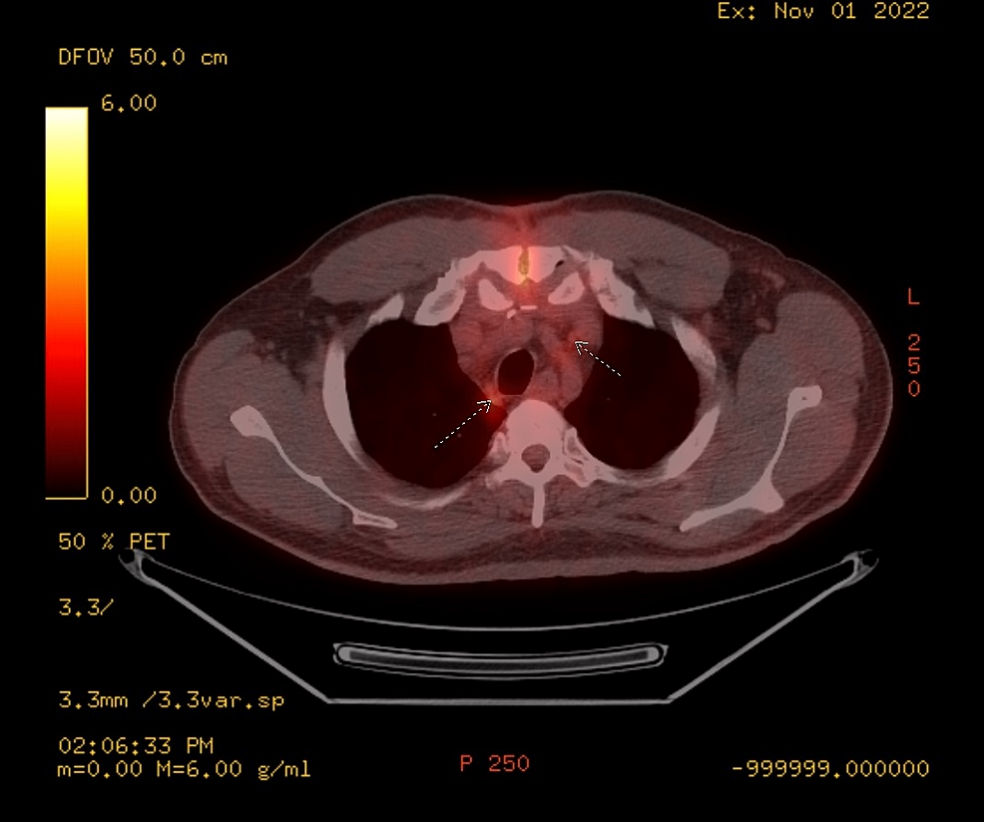
Post-chemotherapy PET/CT scan showing mildly fluorodeoxyglucose (FDG)-avid mediastinal lymph nodes

In August 2022, patient completed one cycle of chemotherapy with paclitaxel 175mg/m2 over three hours on day one, ifosfamide 1200mg/m2 IV over two hours on days one to three, and cisplatin 25mg/m2 over two hours on days one to three (TIP) along with mesna. Eight days after his first cycle of chemotherapy, the patient developed bacteremia with methicillin-sensitive Staphylococcus aureus (MSSA), likely seeded from his left chest wall infusion port, leading to endocarditis and an aortic root abscess. As a result, the patient endured a prolonged hospitalization and underwent aortic valve replacement, debridement of his aorta and ventricles, and bovine pericardial patch placement. He then completed six weeks of IV cefazolin in October 2022. After recovering from his MSSA infection, patient underwent post-chemotherapy PET/CT scan which demonstrated moderate disease progression throughout the abdomen/pelvis, involving pelvic and inguinal lymph nodes, suggestive of chemotherapy failure (Figure 3). There was also a finding of several mildly FDG-avid mediastinal lymph nodes (Figure 4). Patient was initially referred for palliative radiation therapy. However, patient's case was discussed during multi-disciplinary genitourinary tumor board and upon further evaluation, the suspicious thoracic nodes were deemed equivocal for metastases and may possibly be reactive from his recent MSSA infection. Patient was thus planned for adjuvant radiation therapy with curative intent.

**Figure 5:**
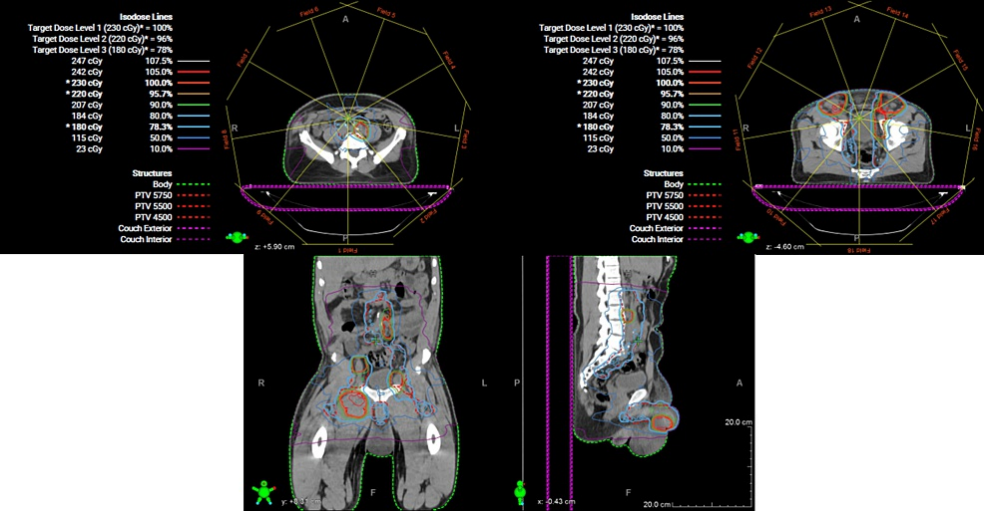
Radiation treatment plan

Initial CT simulation for radiation treatment planning was completed in late October 2022. Patient received 25 fractions over five weeks with simultaneous integrated boost technique, administering 1.8 Gy/fraction to the whole penile shaft, pelvic lymph nodes, and bilateral inguinal lymph nodes, and 2.3 Gy/fraction to gross disease, to achieve 45 Gy and 57.5 Gy, respectively (Figure 5). Two additional fractions of 2.5 Gy were given to areas of gross disease, for a total of 62.5 Gy (EQD2 of 64.15 Gy, assuming an alpha/beta ratio of 10). 

**Figure 6:**
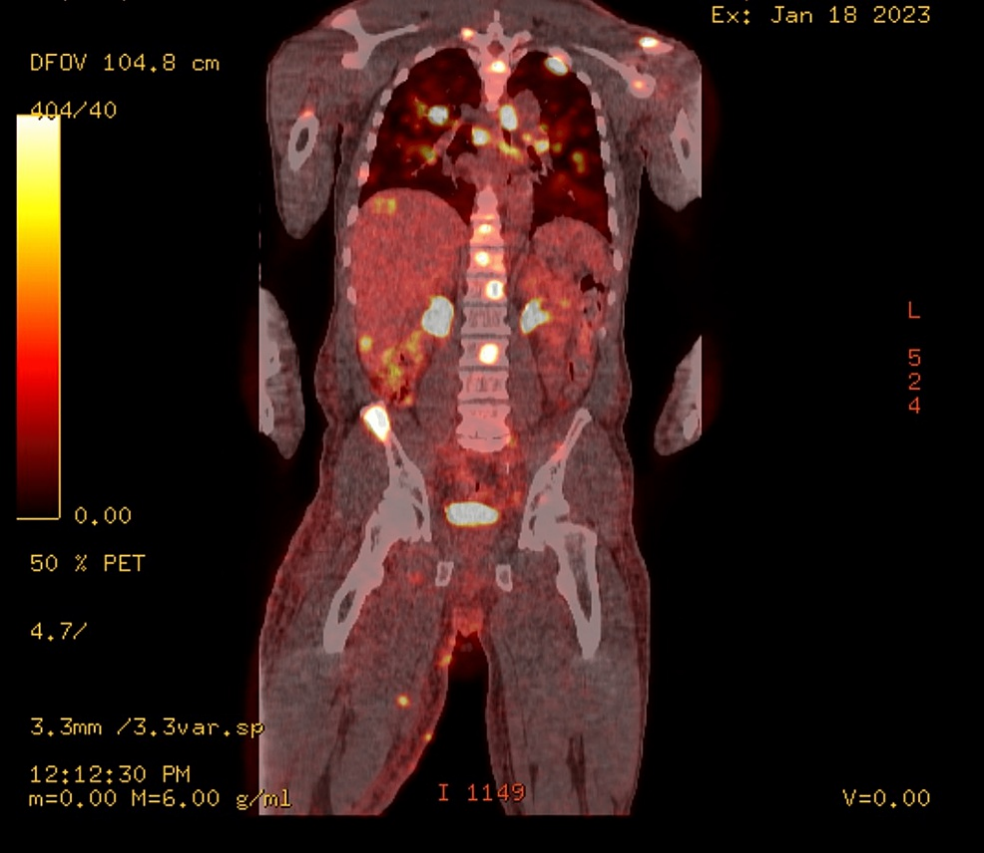
Post-chemotherapy/radiotherapy PET/CT showing new diffuse metastatic disease

One month following radiation therapy, the patient presented at follow-up with swelling and pain in his penis, right groin and testicle. The patient also endorsed new subcutaneous nodules on his right medial thigh. PET/CT scan in January 2023 confirmed metastatic skin deposits in the right medial thigh along with bilateral metastatic pulmonary nodules, hepatic masses, and diffuse osseous metastases (Figure 6). Of note, imaging also showed interval decrease in size and metabolic activity of the irradiated disease in pelvis, penis, and inguinal regions. The patient was then recommended to undergo palliative radiation therapy and additional chemotherapy.

In the following week, the patient received five fractions of radiation therapy (daily fractions of 4 Gy for a total radiation dose of 20 Gy) to his subcutaneous skin nodules. Twelve days following his second radiation treatment, the patient received his second cycle of TIP chemotherapy and was discharged home with no acute concerns. After three weeks, the patient was brought to the emergency department for acute hypoxic respiratory failure. Work-up revealed worsening bilateral pulmonary infiltrates, likely representing pulmonary metastases with possible post-obstructive pneumonia, in addition to non-ST-elevation myocardial infarction (NSTEMI) type 2. Despite aggressive medical management in the ICU, our patient experienced a severe decline in his clinical status and ultimately opted for in-patient hospice, passing away shortly afterwards. 

## Discussion

Penile SCC is a rare but psychologically devastating disease. In most cases, patients do not experience any pain, but first, notice a change in the foreskin or glans. A quarter of patients initially present with enlarged inguinal nodes as well. A biopsy of the lesion, to confirm the diagnosis, and tumor staging is essential for effective treatment planning [2].

Treatment is multimodal and dependent on tumor stage, risk of recurrence, nodal involvement, and advancement of the disease. Nodal involvement and spread are the most significant factors in long-term prognosis. The five-year survival of primary SCC with one positive inguinal lymph node is 79-89%, but metastasis to pelvic nodes is 0 to 17% [2].

For penile carcinoma in situ, topical chemotherapy (5-fluorouracil and imiquimod) is the first-line treatment [4]. In patients with a primary tumor and a low risk of recurrence, limited excision and organ-preserving strategies are preferred so the patient can preserve their penile length and sexual function [3]. Organ-preserving strategies include topical chemotherapy, laser ablation, Mohs micrographic surgery, and RT. For patients with a higher risk of recurrence, penile amputation is considered in order to achieve rapid control [4].

Men with unresectable or locally advanced diseases can receive adjuvant RT, neoadjuvant TIP chemotherapy, or chemoradiation. Radiation therapy can include either external beam radiation or interstitial brachytherapy. Circumcision is required to ensure that the full extent of the tumor receives treatment. High doses of RT to the inguinal and pelvic regions are usually required. Poor prognostic factors in patients who receive RT include a prolonged treatment period (greater than 45 days) and low treatment doses (total dose less than 60 Gy or daily fractions less than 2 Gy) [4].

Early suspicion and diagnosis are important to avoid treatment delays and improve patient outcomes. In this patient’s case, he experienced numerous barriers to appropriate treatment, primarily the unprecedented COVID-19 pandemic. By the time he completed a thorough evaluation, his disease was already found to have metastasized to pelvic lymph nodes which poses a very negative prognostic factor. Not only was his disease aggressive and poorly responsive to first-line systemic therapy, but our patient additionally suffered delays in oncologic treatment because of secondary hospitalizations. Radiographic imaging showed that his cancer responded well to radiotherapy, but his disease was likely already in process of metastasizing in distant organs by the time he underwent local radiation treatment planning. 

## Conclusions

This case is unusual because of its lengthy disease course and obstacles to treatment. Locally advanced penile cancer carries a poor prognosis on its own, and our patient’s diagnosis was not only delayed because of the COVID-19 pandemic, but he endured lengthy hospitalizations for secondary illnesses including endocarditis and myocardial infarction which delayed appropriate therapies. With prompt diagnosis and treatment, this patient may have faced a more positive outcome. 
